# In Vitro Activity of Statins against *Naegleria fowleri*

**DOI:** 10.3390/pathogens8030122

**Published:** 2019-08-08

**Authors:** Aitor Rizo-Liendo, Ines Sifaoui, María Reyes-Batlle, Olfa Chiboub, Rubén L. Rodríguez-Expósito, Carlos J. Bethencourt-Estrella, Desirée San Nicolás-Hernández, Edyta B. Hendiger, Atteneri López-Arencibia, Pedro Rocha-Cabrera, José E. Piñero, Jacob Lorenzo-Morales

**Affiliations:** 1 Instituto Universitario de Enfermedades Tropicales y Salud Pública de Canarias, Universidad de La Laguna, Av. Astrofísico Francisco Sánchez S/N, 38203 Tenerife, Spain; 2 Laboratoire Matériaux-Molécules et Applications, La Marsa, University of Carthage, Carthage 1054, Tunisia; 3 Department of Medical Biology, Medical University of Warsaw, 02091 Warsaw, Poland; 4 Clínica Nivaria, Santa Cruz de Tenerife, Canary Islands, 38203 Tenerife, Spain

**Keywords:** *Naegleria*, therapeutics, statins, fluvastatin, encephalitis

## Abstract

*Naegleria fowleri* causes a deadly disease called primary amoebic meningoencephalitis (PAM). Even though PAM is still considered a rare disease, the number of reported cases worldwide has been increasing each year. Among the factors to be considered for this, awareness about this disease, and also global warming, as these amoebae thrive in warm water bodies, seem to be the key factors. Until present, no fully effective drugs have been developed to treat PAM, and the current options are amphotericin B and miltefosine, which present side effects such as liver and kidney toxicity. Statins are able to inhibit the 3-hydroxy-3-methylglutaryl-coenzyme A (HMG-CoA) reductase, which is a key enzyme for the synthesis of ergosterol of the cell membrane of these amoebae. Therefore, the in vitro activity of a group of statins was tested in this study against two types of strains of *Naegleria fowleri*. The obtained results showed that fluvastatin was the most effective statin tested in this study and was able to eliminate these amoebae at concentrations of 0.179 ± 0.078 to 1.682 ± 0.775 µM depending on the tested strain of *N. fowleri.* Therefore, fluvastatin could be a potential novel therapeutic agent against this emerging pathogen.

## 1. Introduction

Free-living amoebae (FLA) belonging to the *Naegleria* genus are worldwide-distributed protozoa which have predominately been isolated from natural water sources such as lakes, pools and rivers among others [1,2,3,4,5,6,7,8]. *Naegleria fowleri* species, also known as brain-eating amoebae, are the only species of the genus which is able to infect humans, causing a lethal central nervous system (CNS) infection known as primary amoebic meningoencephalitis (PAM) [3,6,8,9,10,11]. Furthermore, the disease mostly affects healthy children and young adults after the nasal epithelium is exposed to untreated water sources, especially during summer, such as lakes, poorly chlorinated swimming pools and/or nose irrigation with contaminated water sources [3,6,8,12,13,14].

The first reported case of PAM was recorded in 1965 in Australia [15]. After this first report, PAM cases have been recorded worldwide, reaching a total of around 440 officially diagnosed cases [16,17]. Currently, the most affected countries are the United States and Pakistan; for example, in the US, 143 cases were reported during the period of 1962–2016 [18].

As mentioned above, *N. fowleri* can infect humans after amoebae-contaminated water enters the nose during water-related activities [3,6,8,12,14]. After that, the amoebae are able to pass through the nasal cavity and penetrate into the olfactory neuroepithelium, migrating through the olfactory nerves to the cribriform plate. Once the cribriform plate is passed, amoebae invade the brain, causing extensive parenchymal inflammation and haemorrhagic necrosis [3,6,9,10,19]. It is also important to mention that PAM is characterised as a rapid and fulminant disease with non-specific clinical symptoms. The average time of symptom appearance after exposure is 1–9 days (median 5 days) after exposure to contaminated water sources, whereas the average patient death is 1–18 days (median 10 days) after symptoms begin [4,7,17,19,20].

Moreover, diagnosis is often performed *post-mortem* because of the clinical symptoms and course of the disease mentioned above. Among the most common symptoms are temperature, seizures, stiff neck, severe bi-frontal headaches and coma in the later stages of the disease [3,4,7,19,21].

Regarding treatment of PAM when diagnosed, current therapy involves a combination of amphotericin B and other drugs such as azithromycin, rifampin, azoles and, lately, miltefosine [3,9,10,20,22,23]. The addition of miltefosine to this drug combination as well as hypothermia have recently resulted in the successful survival of treated patients [9,24,25,26,27]. However, the treatment is frequently associated with severe adverse effects such as renal toxicity, anaemia, nausea, vomiting or even brain damage. Worryingly, the mortality as a consequence of PAM is above 95–97% of registered cases [3,21,28,29]. According to these data, there is an urgent need to develop new anti-*Naegleria* agents to treat PAM quickly and efficiently while also causing low toxicity.

The 3-hydroxy-3-methylglutaryl-coenzyme A (HMG-CoA) reductase is an enzyme that regulates the mevalonate pathway, which is the metabolic way of producing cholesterol in humans and ergosterol in fungi, plants and protozoa. Ergosterol, 7-dehydrostigmaterol (7DHC) and cholesterol have been reported as the main sterols in *N. fowleri*, which is the first, essential for the integrity of cell membranes [30]. Among known commercially available inhibitors of the HMG-CoA reductase, statins are a group of molecules widely used to lower cholesterol levels in patients. Moreover, their activity against another FLA was recently demonstrated showing promising results [23,31,32].

Therefore, the in vitro activities of six statins (simvastatin, fluvastatin, atorvastatin, pravastatin, mevastatin and lovastatin) were evaluated against the trophozoite stage of *N. fowleri*, using a colorimetric method based on alamarBlue^®^ in comparison with the reference drug Amphotericin B (Amph B). In addition to the activity assays, an in vitro toxicity assay was also performed [32,33,34].

## 2. Results

### 2.1. In Vitro Activity of the Tested Statins against the Trophozoite Stage of Naegleria fowleri

In this study, the activity of six statins was tested against two strains of *N. fowleri*. All the tested compounds were active against the tested amoebic strains. However, atorvastatin, fluvastatin, simvastatin and lovastatin showed amoebicidal effects, whereas pravastatin only induced amoebostatic effects. Moreover, in the case of mevastatin, this compound was only active against the *N. fowleri* ATCC 30215 strain (Table 1).

**Figure 1:**
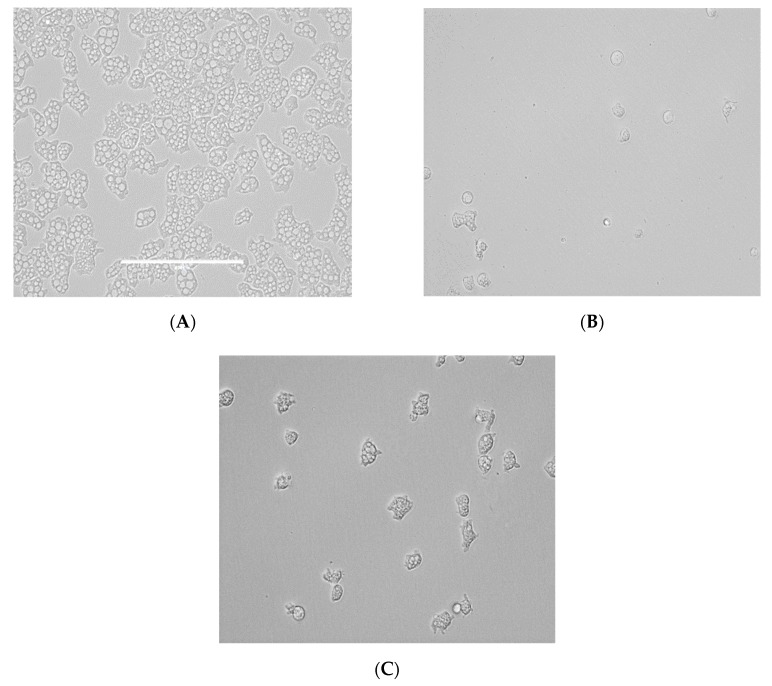
Growth inhibition of *N. fowleri* (ATCC 30808) trophozoites at 48 h. Negative control (**A**), fluvastatin 3.125 µM (**B**) and fluvastatin 0.195 µM (**C**). All images (×40) are representative of the population of treated amoeba and are based on the live cell imaging microscope EVOS FL cell imaging system.

**Figure 2:**
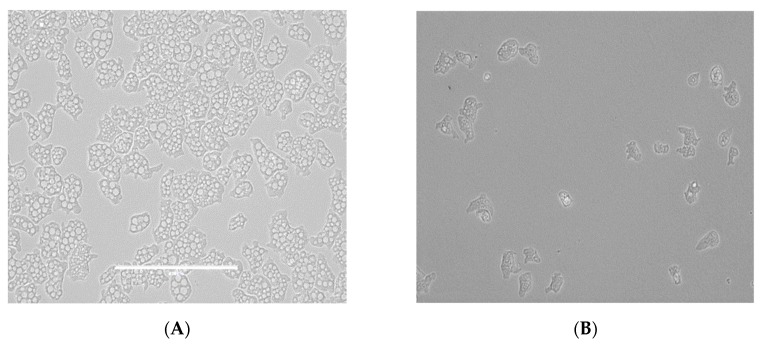

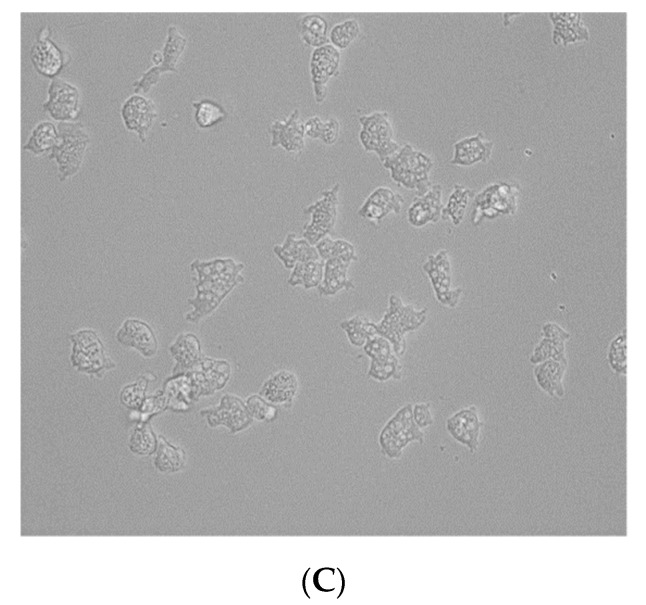
Growth inhibition of *N. fowleri* (ATCC 30808) trophozoites at 48 h. Negative control (**A**), atorvastatin 12.5 µM (**B**) and atorvastatin 3.125 µM (**C**). All images (×40) are representative of the population of treated amoeba and are based on the live cell imaging microscope EVOS FL cell imaging system.

**Figure 3:**
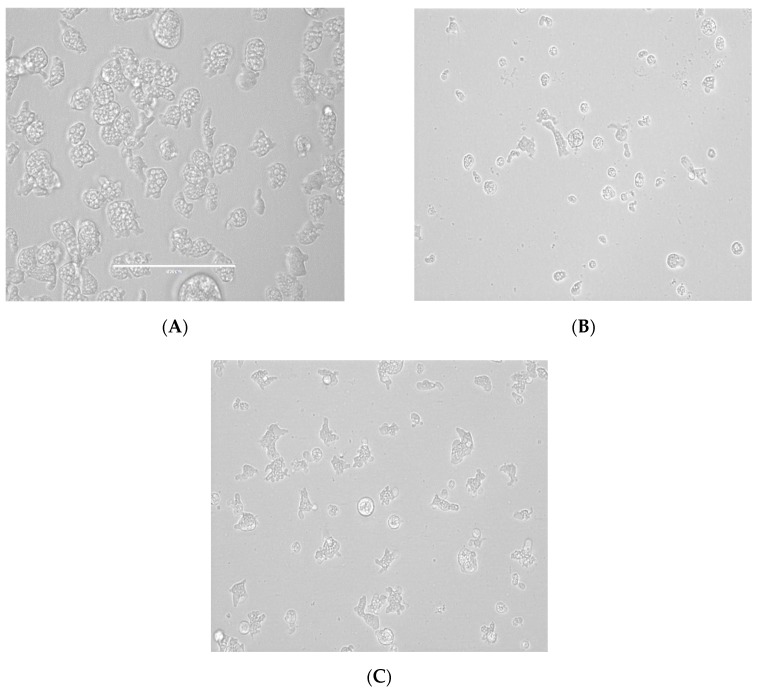
Growth inhibition of *N. fowleri* (ATCC 30215) trophozoites at 48 h. Negative control (**A**), fluvastatin 12.5 µM (**B**) and fluvastatin 3.125 µM (**C**). All images (×40) are representative of the population of treated amoeba and are based on the live cell imaging microscope EVOS FL cell imaging system.

**Figure 4:**
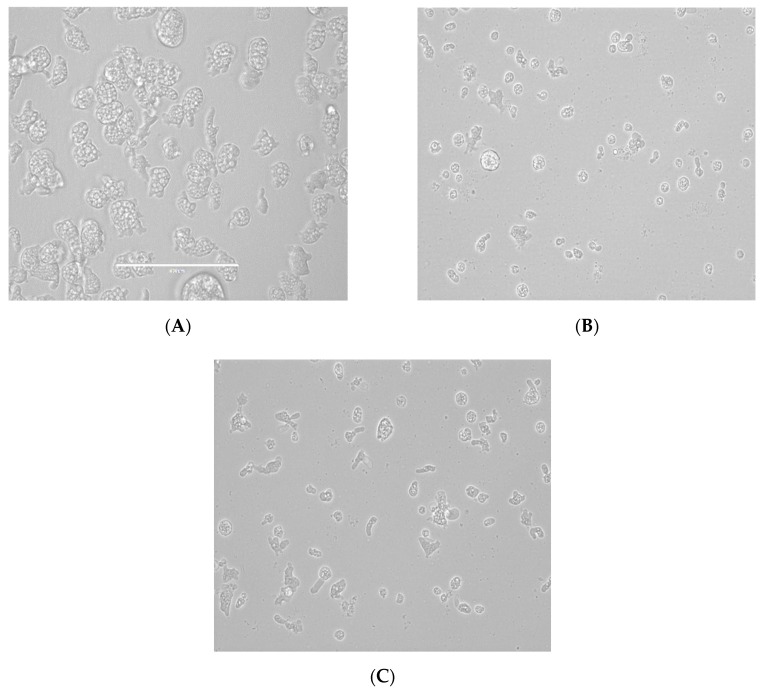
Growth inhibition of *N. fowleri* (ATCC 30215) trophozoites at 48 h. Negative control (**A**), atorvastatin 25 µM (**B**) and atorvastatin 6.25 µM (**C**). All images (×40) are representative of the population of treated amoeba and are based on the live cell imaging microscope EVOS FL cell imaging system.

From all the tested statins, fluvastatin was the most active one showing IC_50_ values ranging from 0.179 ± 0.078 to 1.682 ± 0.775 µM depending on the tested strain of *N. fowleri.* Furthermore, atorvastatin also showed high activity values between 6.278 ± 1.085 to 7.629 ± 0.696 µM. Moreover, strong changes in morphology as well as in the number of intracellular vesicles were observed in the two strains of *Naegleria fowleri* when they were incubated with serial dilutions of atorvastatin and fluvastatin especially (Figure 1, Figure 2, Figure 3 and Figure 4).

### 2.2. In Vitro Toxicity against Murine Macrophages

The toxicity of the tested compounds was determined in vitro against the J774A.1 murine macrophage cell line. The less toxic molecules were fluvastatin and atorvastatin with CC_50_ values between 100 and 1000 times higher than the IC_50_ values obtained in the case of fluvastatin (Table 1 and Table 2).

## 3. Discussion

The number of encephalitis cases due to *Naegleria fowleri* has been increasing worldwide. Moreover, as PAM is a highly lethal infection, there is an urgent need to develop novel antiamoebic agents which are able to eliminate the pathogen in a fast and highly effective way [6].

Statins have been previously tested in another pathogenic genus of FLA in our laboratory, *Acanthamoeba*, showing that atorvastatin, simvastatin and fluvastatin were able to eliminate both life cycle stages of *Acanthamoeba* [30,31]. Furthermore, the enzyme 3-hydroxy-3-methylglutaryl–coenzyme A (HMG-CoA) reductase is the main target of these family of molecules, and, thus, it is suspected that the same enzyme is present in *Naegleria fowleri* and is also inhibited by these drugs. Moreover, the same enzyme is widely expressed in vertebrates and other parasitic protozoa, and the active site of this enzyme has been reported previously to be highly conserved from an evolutionary point of view [31,32].

**Figure 5:**
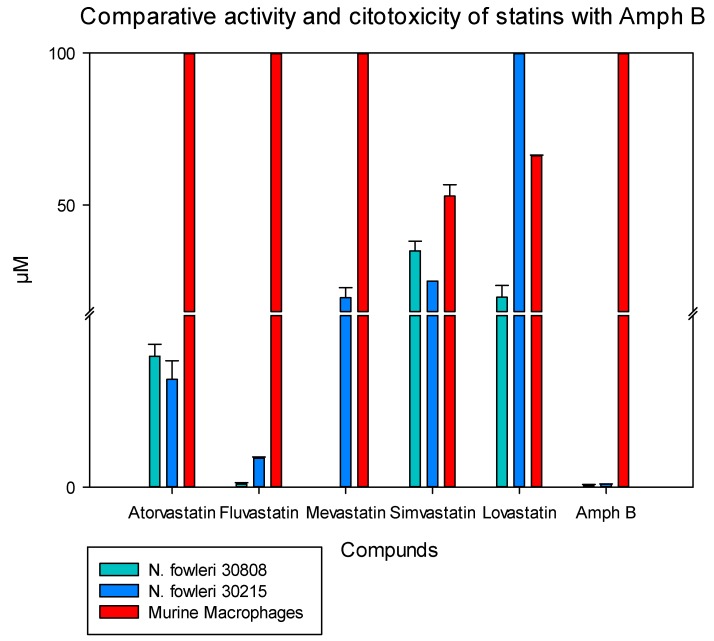
Comparison of the evaluated activity of the statins used in this study against two strains of *N. fowleri* (ATCC 30808, ATCC 30215) and the induced cytotoxicity in the murine macrophage cell line (ATCC TIB-67); the reference drug amphotericin B was included as a positive control and to compare the obtained values.

As it has been previously reported in FLA, the main sterol membrane components are ergosterol and 7-dehydrostigmaterol (7DHC), apart from cholesterol in the case of *N. fowleri*. Moreover, ergosterol is essential for the integrity of the cell membrane in this species [30]. In our study, we identified some statins which were able to eliminate trophozoites belonging to two different types of *N. fowleri* strains in a highly effective dose, causing low toxic side effects (Table 1 and Table 2 and Figure 1, Figure 2, Figure 3 and Figure 4). Therefore, statins, at least in vitro, could be a potential family of therapeutic agents against this emerging pathogen. Another important fact to highlight is that statins are able to penetrate the blood–brain barrier [31,32] and, thus, if future studies in vivo support the in vitro data, at least fluvastatin and atorvastatin are supported by our results presented in this study (Figure 5).

However, because of the very serious nature of PAM, higher statin levels may be used, and any side effects must be accepted and lessened to some extent and compensated for by dietary uptake [31,32]. Moreover, the bioavailability of statins differs greatly, ranging from 5% to 60%, as reported previously with the elimination of half-lives ranging from 1 h for fluvastatin to 19 h for rosuvastatin [35,36]. In a previous clinical assay, using simvastatin for the long-term treatment of two weeks allowed the researchers to check for CSF (Cerebrospinal fluid) biomarkers, showing a reduction in some of them in the statin-treated group [37].

Additionally, further experiments should be carried out in order to confirm whether statins act differently at different temperatures, as this does not seem to have been investigated. From the obtained data, we are able to conclude that the mentioned statins are as effective as the current drug being used to treat PAM, and to the best of our knowledge, lower costs and side effects support further developments to propose statins as a novel effective therapeutic agent against *N. fowleri* infections.

## 4. Materials and Methods

### 4.1. Amoebic Cultures

To test the amoebicidal activity of the compounds, two strains of *Naegleria fowleri* (ATCC^®^ 30808™ and ATCC^®^ 30215™) of the American Type Culture Collection (LG Promochem, Barcelona, Spain) were used. The strain was axenically cultured at 37 °C in 2% (*w/v*) Bactocasitone medium (Thermo Fisher Scientific, Madrid, Spain) supplemented with 10% (*v/v*) foetal bovine serum (FBS), containing 0.5 mg/mL of streptomycin sulfate (Sigma-Aldrich, Madrid, Spain) and 0.3 µg/mL of Penicillin G Sodium Salt (Sigma-Aldrich, Madrid, Spain). Strains were kept in the biological security facilities level 3 of our institution following Spanish biosafety guidelines for this pathogen.

For the toxicity assays, the murine macrophage J774A.1 (ATCC # TIB-67) cell line was cultured in Dulbecco’s Modified Eagle’s medium (DMEM, w/v), supplemented with 10% (v/v) fetal bovine serum and 10 µg/mL gentamicin (Sigma-Aldrich, Madrid, Spain), at 37 °C and 5% CO_2_ atmosphere. For the experiments, all the strains were used during the logarithmic phase of growth.

### 4.2. Chemicals

A total of six statins were used in this study. The statins were purchased from Cayman Chemicals (Vitro SA, Madrid, Spain) and included atorvastatin, fluvastatin, simvastatin, pravastatin, mevastatin and lovastatin. The stock solutions for the experiments were prepared in dimethyl sulfoxide (DMSO) and were maintained at −20 °C until required for the experiments. As a positive control, amphotericin B was used.

### 4.3. In Vitro Activity Assays against the Trophozoite Stage of Naegleria fowleri

The activity of the tested statins against the trophozoite stage of *Naegleria fowleri* was determined in vitro using a modified colorimetric assay based on the oxido-reduction of alamarBlue^®^ reagent (Life Technologies, Barcelona, Spain), as previously described [38,39].

Briefly, the trophozoites were counted using a Countess II FL automatic cell counter (Thermo Fisher Scientific, Madrid, Spain) to prepare a working cell suspension (10^5^ cells/well), and 50 µL per well was added in a 96-well plate (Thermo Fisher Scientific, Madrid, Spain).

After that, a serial dilution of the different statins diluted in the same culture medium was added to the plate (50 µL) (in all tests, 2% DMSO was used to dissolve the highest dose of the compounds without inducing any effects on the parasites). As a negative control, the trophozoites were incubated with the medium alone. Finally, the alamarBlue^®^ reagent was positioned into each well (10% of medium volume) and plates were incubated with slight agitation for 48 h at 37 °C. Subsequently, the plates were analysed with an EnSpire^®^ Multimode Plate Reader (Perkin Elmer, Madrid, Spain) using a wavelength of 570 nm and a reference wavelength of 630 nm. To calculate the percentages of growth inhibition and 50% inhibitory concentrations (IC_50_), a non-linear regression analysis was performed with a 95% confidence limit using the SigmaPlot 12.0 software (Systat Software Inc., London, UK). All the experiments were performed in triplicate and the mean values were also calculated. A paired two-tailed *t*-test was used for the analysis of the data and the values of *p* < 0.05 were considered statistically significant.

### 4.4. Cytotoxicity Assays

To evaluate the toxicity of the statins used in this study, the murine macrophage cell line J774A.1 (ATCC TIB-67) was used. First, the macrophages were cultured in RPMI 1640 medium without phenol red (Roswell Park Memorial Institute, Thermo Fisher Scientific Inc., Waltham, MA, USA). After that, the cells were seeded (50 µL) in a 96-well plate (10^5^ cells/mL) and serial dilutions (diluted in medium) of the statins were added (50 µL) to reach a final volume of 100 μL/well, as previously described [38,39]. A negative control was used, consisting of cells, and was incubated with the medium alone. Finally, the alamarBlue^®^ reagent was placed into each well (10% of final volume) and incubated for 24 h at 37 °C and 5% CO_2_ atmosphere.

The plates were analysed with an EnSpire^®^ Multimode Plate Reader, as mentioned above. The 50% of cytotoxic values (CC_50_) were calculated using the statistical analysis software SigmaPlot 12.0, as previously reported. All the experiments were performed three times and the mean values calculated. Finally, the selectivity index was calculated based on the obtained IC_50_ and the CC_50_, as shown in Table 2.

## 5. Conclusions

This study assesses the in vitro efficacy of statins against *Naegleria fowleri*. Although rare, infections due to this pathogen are usually lethal and, therefore, new effective treatments are needed. The obtained results have highlighted that at least fluvastatin could be a potential new candidate for the treatment of PAM. Further in vivo studies should be developed to fully establish this compound as a novel therapeutic agent against PAM.

## Figures and Tables

**Table 1:** Activity of the evaluated statins against the trophozoite stage of *Naegleria fowleri* and cytotoxicity against the J774A.1 macrophage cell line.

Compound (µM)	Structure	*N. fowleri* ATCC 30808 IC_50_ (µM)	*N. fowleri* ATCC 30215 IC_50_ (µM)	Murine Macrophages CC_50_ (µM)
Atorvastatin	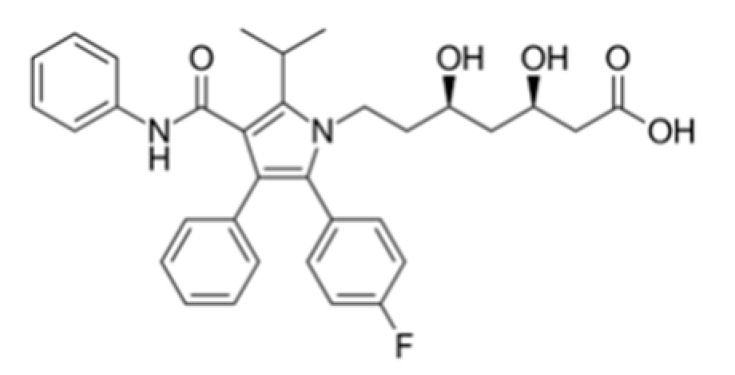	7.629 ± 0.696	6.278 ± 1.085	≥200
Fluvastatin	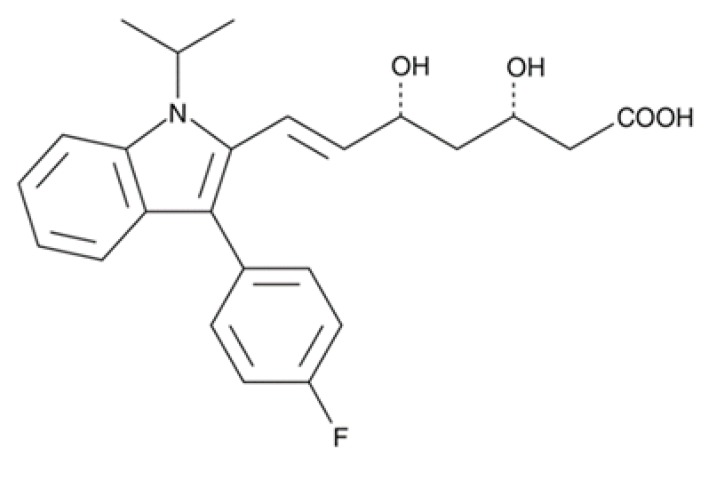	0.179 ± 0.078	1.682 ± 0.775	≥200
Pravastatin	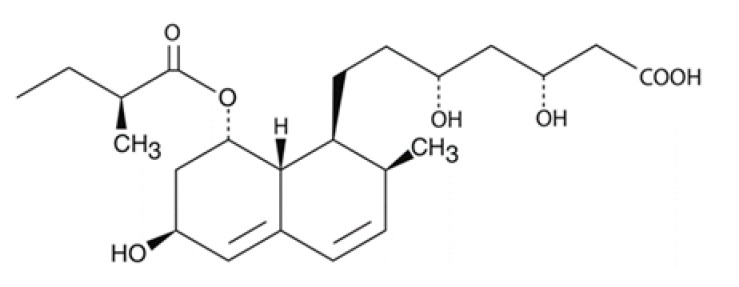	Amoebostatic	Amoebostatic	≥100
Mevastatin	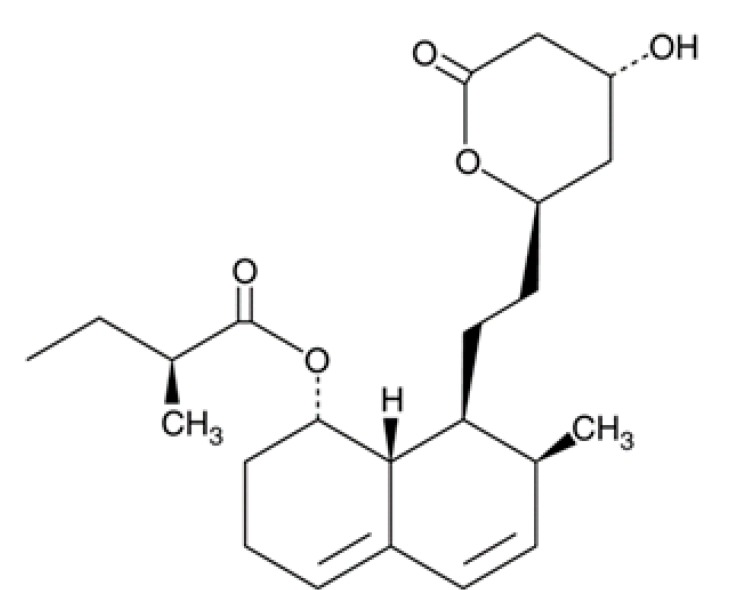	Amoebostatic	19.542 ± 3.295	≥200
Simvastatin	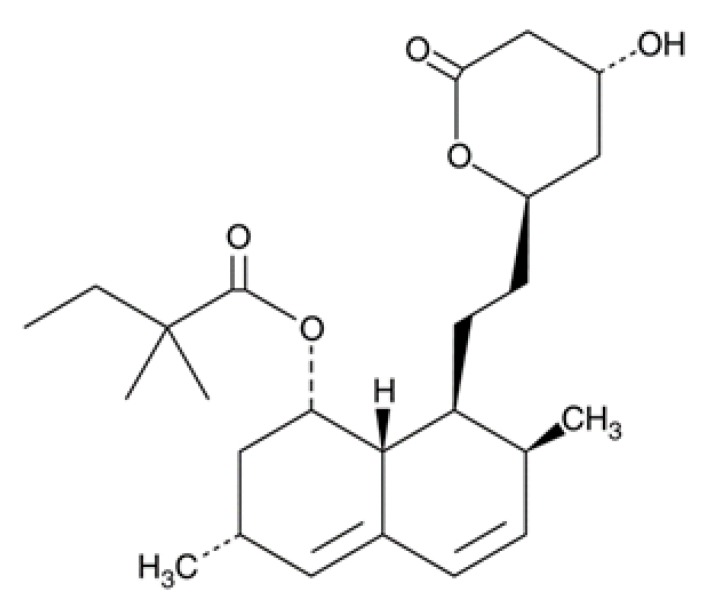	34.943 ± 3.149	≈25	52.971 ± 3.691
Lovastatin	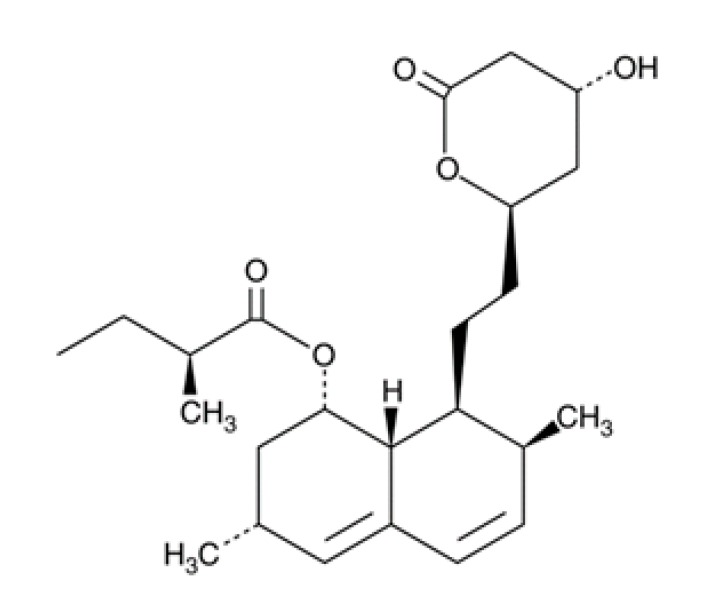	19.742 ± 3.824	≥100	66.170 ± 0.268
Amphotericin B	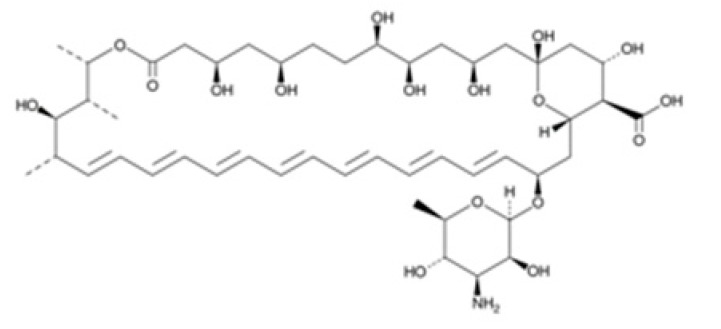	0.121 ± 0.032	0.166 ± 0.026	≥200

**Table 2:** Selectivity index (CC_50_/IC_50_) of the tested statins evaluated in this study and the positive control amphotericin B. ND indicates non-determined.

Selectivity Index (CC_50_/IC_50_)		
Compounds (µM)	ATCC 30808	ATCC 30215
Atorvastatin	≥26.215	≥31.857
Fluvastatin	≥1117.318	≥118.906
Pravastatin	ND	ND
Mevastatin	ND	≥10.234
Simvastatin	≥1.6197	≈2.264
Lovastatin	≥3.3517	ND
Amphotericin B	≥1652.893	≥1204.819
